# Topographic analysis of vascular changes in retrograde trans-synaptic degeneration

**DOI:** 10.1371/journal.pone.0337283

**Published:** 2025-12-02

**Authors:** Yeji Moon

**Affiliations:** Department of Ophthalmology, Asan Medical Center, University of Ulsan College of Medicine, Seoul, Republic of Korea; University of South Florida, UNITED STATES OF AMERICA

## Abstract

**Objectives:**

To investigate the vascular signatures of retrograde trans-synaptic degeneration (RTSD) on OCT and OCTA, and to assess whether these changes differ from those associated with non-arteritic anterior ischemic optic neuropathy (NAION).

**Methods:**

This retrospective cohort study included 24 eyes with RTSD and 12 eyes with NAION. Retinal regions corresponding to visual field defects were topographically defined and compared with anatomically matched internal control regions from the fellow eye. Structural (peripapillary retinal nerve fibre layer(pRNFL), macular ganglion cell–inner plexiform layer(mGCIPL)) and vascular (peripapillary vessel density(pVD], macular vessel density(mVD)) parameters were calculated as affected-to-control ratios (%). Spearman correlation and ANCOVA were used to evaluate structure–vascular relationships and compare regression slopes between groups.

**Results:**

Both groups exhibited significant structural and vascular reductions in affected regions. NAION eyes demonstrated greater reductions in pRNFL, pVD, and mVD ratios compared to RTSD (all *p* < 0.05), while mGCIPL ratios showed a non-statistically significant difference (*p* = 0.065). The correlation between mGCIPL and mVD was higher in NAION (ρ = 0.815, *p* = 0.001) than in RTSD (ρ = 0.469, *p* = 0.021). Age-adjusted ANCOVA with an interaction term demonstrated that the slope of the relationship between structural thinning and vascular loss was significantly more pronounced in NAION (interaction β = –0.75, *p* = 0.049).

**Conclusions:**

RTSD demonstrated relatively modest vascular compromise compared to NAION, which is presumed to reflect secondary changes due to reduced metabolic demand following retrograde neuronal degeneration. Quantitative structure–vascular analysis using RTSD as a reference model may aid in distinguishing secondary degeneration from primary ischemic injury in optic neuropathies.

## Introduction

Retrograde trans-synaptic degeneration (RTSD) refers to the progressive loss of retinal neurons secondary to damage in the postgeniculate visual pathway, such as infarction or tumor involving the occipital cortex or optic radiations [1–3]. Optical coherence tomography (OCT) enables in vivo visualization of corresponding retinal structural thinning, particularly in the macular ganglion cell–inner plexiform layer (mGCIPL) and peripapillary retinal nerve fiber layer (pRNFL) [4–7].

More recently, optical coherence tomography angiography (OCTA) has emerged as a non-invasive tool for assessing retinal microvasculature. While OCTA findings have been well-characterized in primary optic neuropathies such as non-arteritic anterior ischemic optic neuropathy (NAION), in which ischemic vascular compromise underlies the pathogenesis, its application in RTSD remains limited [8–10]. Only limited studies have reported reduced vessel density in RTSD-affected eyes, but the extent and significance of these vascular changes relative to structural degeneration are not fully understood [11,12].

Given that RTSD represents secondary neurodegeneration and NAION reflects primary ischemic injury, comparing OCTA-based vascular alterations in these two conditions may provide insights into their distinct pathophysiological mechanisms. Moreover, investigating the relationship between retinal structural loss and vascular compromise may help differentiate ischemic from trans-synaptic processes.

The purpose of this study was to compare OCT and OCTA findings between RTSD and NAION, and to evaluate the correlation between structural and vascular changes in each condition.

## Materials and methods

This retrospective cohort study adhered to the tenets of the Declaration of Helsinki and was approved by the Institutional Review Board (IRB) of Asan Medical Center (IRB No. S2024-0155–0001). Due to the retrospective nature of the study, the requirement for informed consent was waived.

### Study design and participants

This retrospective comparative study included patients diagnosed with either RTSD or NAION who visited the neuro-ophthalmology clinic at Asan Medical Center between January 2022 and December 2023. Patients in the RTSD group had visual field (VF) defects associated with postgeniculate visual pathway lesions involving the optic radiation or primary visual cortex, with OCT and OCTA performed at least four years after lesion onset. Patients in the NAION group had a clinical diagnosis of unilateral NAION with inferior altitudinal VF defects and underwent OCT/OCTA imaging at least six months after onset to allow structural stabilization. A minimum interval of four years after lesion onset was applied for RTSD to include only cases in the stable chronic stage, as retinal thinning after postgeniculate lesions typically plateaus beyond three years [6]. For NAION, patients were included ≥ 6 months after onset to represent the chronic post-ischemic phase following resolution of disc edema [13,14]. These differing intervals ensured that both groups were evaluated at comparable stable phases of disease evolution, minimizing the influence of acute-stage changes.

All participants received comprehensive ophthalmic evaluations, and anonymized clinical data—including best-corrected visual acuity (BCVA), results of VF testing, OCT, and OCTA—were collected. Automated perimetry was performed using the Humphrey Field Analyzer with the SITA-Standard 30−2 program (Carl Zeiss Meditec, Dublin, CA, USA), and VF parameters included the visual field index (VFI) and mean deviation (MD). Data were accessed from 20/03/2024 to 30/08/2024, we obtained the clinical data which were anonymized. Patients were excluded if they were under 18 years of age, had confirmed elevated intracranial pressure or papilledema, or had systemic, intracranial, or ocular conditions that could confound visual function or interfere with OCT/OCTA measurements.

### OCT/OCTA image acquisition and processing

OCT images were obtained using the Cirrus HD-OCT system (Carl Zeiss Meditec, Dublin, CA, USA). Images with signal strength ≥ 6 were included, and all scans were manually reviewed for segmentation accuracy by one author (Y.M.). pRNFL thickness was automatically measured along a 3.46-mm diameter circumpapillary circle in the temporal, superior, nasal, and inferior quadrants. mGCIPL thickness was measured within an elliptical annulus centered on the macula (inner ring: 1.0 mm × 1.2 mm; outer ring: 4.0 mm × 4.8 mm), divided into six sectors: superotemporal, superior, superonasal, inferonasal, inferior, and inferotemporal. For RTSD, mGCIPL values were averaged across the temporal (superotemporal and inferotemporal) or nasal (superonasal and inferonasal) sectors according to the side of the visual field defect, to represent the affected hemiretina corresponding to the postgeniculate lesion. For NAION, the affected mGCIPL was calculated as the mean of the superotemporal, superior, and superonasal sectors, reflecting the typical superior retinal involvement associated with inferior visual field loss.

OCTA images were acquired using the AngioVue OCTA system (Optovue Inc., Fremont, CA, USA), including a 4.5 × 4.5-mm scan centered on the optic disc and a 6 × 6-mm scan centered on the fovea. Images with a signal strength index < 45, significant motion artifacts, or segmentation errors were excluded. Peripapillary vessel density (pVD) was measured within a 750-μm annulus extending from the optic disc margin in the radial peripapillary capillary layer. Eight pVD sectors (temporosuperior, superotemporal, superonasal, nasosuperior, nasoinferior, inferonasal, inferotemporal, and temporoinferior) were averaged to produce quadrant-wise values in the temporal, superior, nasal, and inferior quadrants. Macular vessel density (mVD) was assessed in the superficial capillary plexus, separately for parafoveal (1.0–3.0 mm annulus) and perifoveal (3.0–6.0 mm annulus) zones, with vessel densities calculated in four quadrants (temporal, superior, nasal, inferior) for each.

### Structural and vascular parameter measurement

**Fig 1:**
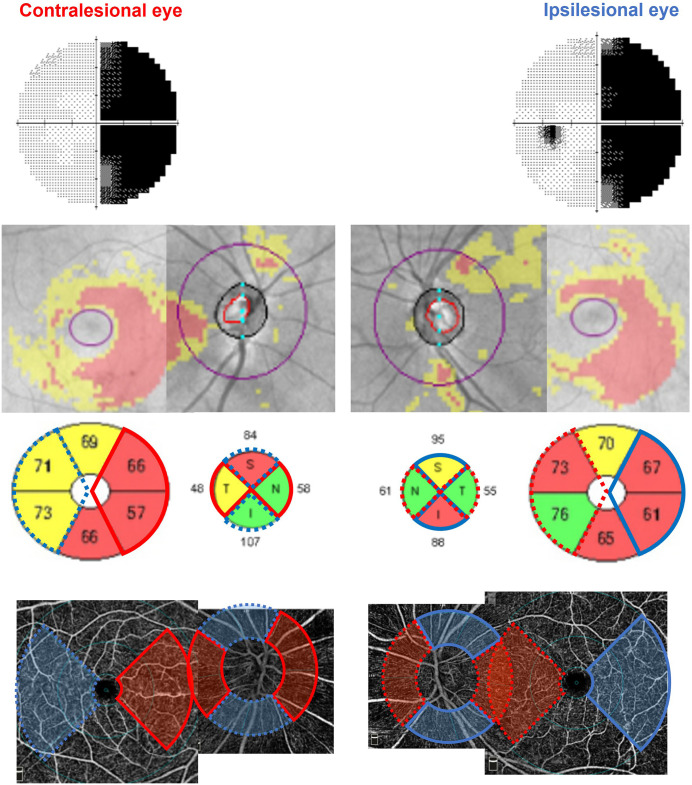
Region selection for structural and vascular analysis in RTSD. In a patient with RTSD after a post-chiasmal lesion, the affected regions were defined according to the homonymous VF defect and included the nasal macular hemiretina and nasal/temporal pRNFL of the contralesional eye (red solid sectors) and the temporal macular hemiretina and superior/inferior pRNFL of the ipsilesional eye (blue solid sectors). The topographically matched but unaffected regions (dotted sectors) in each eye served as internal controls for normalization.

The RTSD-affected area was defined based on the pattern of VF defect. For instance, in patients with right homonymous hemianopia due to a left hemisphere lesion, the affected regions included the nasal macular hemiretina and nasal and temporal pRNFL of the right (contralesional) eye and the temporal macular hemiretina and superior and inferior pRNFL of the left (ipsilesional) eye. The mean values of pRNFL, mGCIPL, pVD, and mVD within these RTSD sectors were calculated for each eye. The internal controls were selected from topographically matched regions: the nasal macular hemiretina of the ipsilesional eye served as the control for the contralesional nasal hemiretina, and the temporal macular hemiretina of the contralesional eye served as the control for the ipsilesional temporal hemiretina (Fig 1).

**Fig 2:**
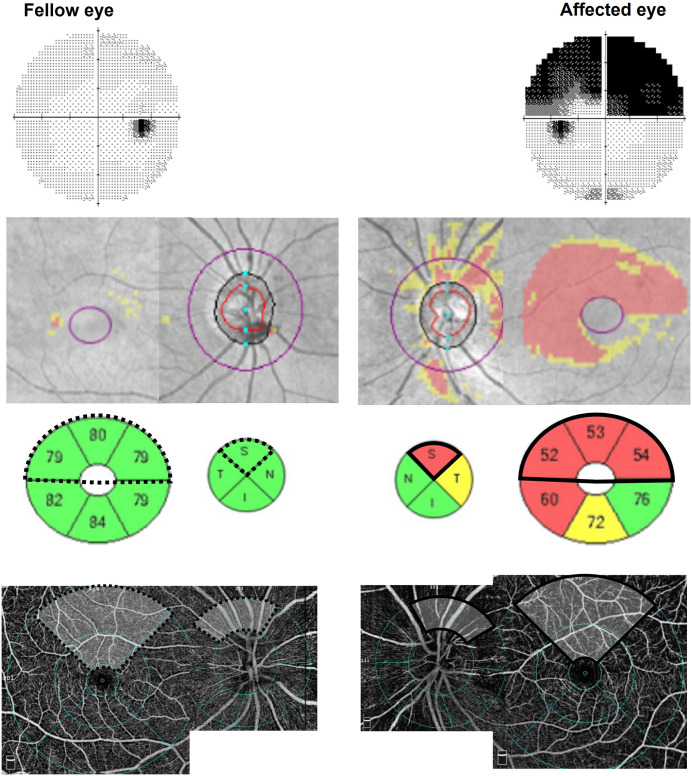
Region selection for structural and vascular analysis in NAION. In a patient with NAION, the affected region (solid black sector) included the superior macula and superior pRNFL of the affected eye, corresponding to an inferior VF defect, while the dotted black sector in the fellow eye represented the topographically matched internal control region. Ratio values (%) were calculated as affected/ control × 100 for both structural (pRNFL and mGCIPL, *μ*m) and vascular (pVD and mVD, %) parameters to normalize inter-individual variability.

In the NAION group, the affected region was defined as the superior macula and superior pRNFL of the involved eye in patients with inferior VF defects. The control region was the corresponding superior macular region of the contralateral, unaffected eye. (Fig 2)

Fellow eyes were used as internal controls to minimize interindividual variability in ocular anatomy and systemic microvascular status. For RTSD, only unilateral postgeniculate lesions with clearly lateralized hemianopic field defects were included, ensuring no contralateral hemiretinal involvement. For NAION, only unilateral cases without clinical or imaging evidence of subclinical fellow-eye involvement were analyzed. This approach provides a physiologically valid internal reference while avoiding intersubject confounding.

To account for interindividual variability, ratio values were calculated for each structural and vascular parameter as the measurement in the affected region divided by that in the control region, multiplied by 100 to express as a percentage. This within-subject normalization allowed for quantification of localized damage relative to internal controls.

### Statistical analysis

All statistical analyses were performed using SPSS software (version 23.0; IBM Corp., Armonk, NY, USA). Continuous variables are presented as mean ± standard deviation. Between-group differences in continuous variables were evaluated using the Mann–Whitney U test, and comparisons between affected and control regions within each group were performed using the Wilcoxon signed-rank test. Correlation analyses between structural parameters (pRNFL, mGCIPL) and vascular parameters (pVD, mVD) were performed separately for each group using Spearman rank correlation. To compare the relationship between structural and vascular changes across groups, an analysis of covariance (ANCOVA) was conducted using a linear regression model including an interaction term. A significant interaction was interpreted as evidence of differing regression slopes across groups. A *p*-value < 0.05 was considered statistically significant.

## Results

The RTSD group was significantly younger than the NAION group (44.1 ± 16.3 vs. 62.8 ± 11.2 years, *p* = 0.007). There was no significant difference in the proportion of male patients between groups (33.3% in RTSD vs. 58.3% in NAION, *p* = 0.414). Baseline BCVA was significantly better in the RTSD group (0.05 ± 0.09 logMAR) compared to the NAION group (0.35 ± 0.36 logMAR, *p* = 0.001). No significant differences were found in baseline VF test results between groups: VFI was 68.8 ± 20.3% in RTSD and 71.2 ± 17.0% in NAION (*p* = 0.625), and MD was –11.85 ± 6.77 dB in RTSD and –9.97 ± 5.65 dB in NAION (*p* = 0.471).

Table 1 presents the comparison of structural and vascular parameters between affected and control retinal regions within each disease group. In both NAION and RTSD eyes, significant reductions were observed in the affected regions compared to their respective topographically matched control regions. This pattern was consistent across all evaluated parameters, including pRNFL thickness, mGCIPL thickness, peripapillary vessel density (pVD), and macular vessel density (mVD).

**Table 1:** Comparison of structural and vscular parameters between affected and control retinal regions in RTSD and NAION groups.

	Affected region	Control region	*p*-value
RTSD (n = 24 eyes)			
pRNFL thickness, *μ*m	80.3 ± 26.5	88.9 ± 28.2	**< 0.001**
mGCIPL thickness, *μ*m	60.1 ± 10.5	79.8 ± 6.0	**< 0.001**
Peripapillary VD, %	45.2 ± 5.2	48.4 ± 3.5	**< 0.001**
Macular VD, %	43.4 ± 5.8	48.9 ± 4.2	**0.006**
NAION (n = 12 eyes)			
pRNFL thickness, *μ*m	73.0 ± 19.5	122.0 ± 24.0	**< 0.001**
mGCIPL thickness, *μ*m	56.8 ± 5.8	84.42 ± 7.2	**< 0.001**
Peripapillary VD, %	31.9 ± 9.2	52.6 ± 6.3	**< 0.001**
Macular VD, %	42.0 ± 5.4	51.84 ± 4.17	**0.003**

To further quantify and compare the degree of retinal involvement across groups, we calculated the ratio of each affected parameter relative to its control value, expressed as a percentage (Table 2). Both structural ratios (pRNFL and mGCIPL) and vascular ratios (pVD and mVD) were lower in the NAION group than in the RTSD group. Groupwise comparisons revealed statistically significant differences for pRNFL, pVD, and mVD (all p < 0.05), while the difference in mGCIPL did not reach statistical significance (*p* = 0.065).

**Table 2:** Comparison of affected-to-control ratios between RTSD and NAION groups.

	RTSD (n = 24 eyes)	NAION (n = 12 eyes)	*p*-value
pRNFL thickness ratio, %	90.5 ± 8.5	60.9 ± 14.3	**< 0.001**
mGCIPL thickness, ratio %	75.4 ± 12.4	67.7 ± 8.9	0.056
Peripapillary VD ratio, %	88.8 ± 9.8	61.0 ± 18.7	**< 0.001**
Macular VD ratio, %	92.9 ± 11.7	81.6 ± 13.2	**0.034**

### Correlation between structural and vascular measurements

**Fig 3:**
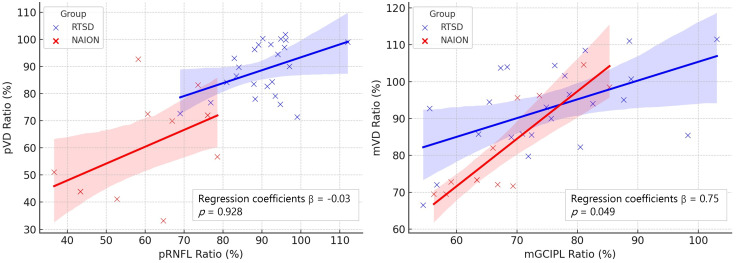
Scatterplots demonstrating the relationship between structural and vascular ratios in eyes with retrograde trans-synaptic degeneration (RTSD, blue) and non-arteritic anterior ischemic optic neuropathy (NAION, red). (A) Peripapillary retinal nerve fiber layer (pRNFL) ratio versus peripapillary vessel density (pVD) ratio. (B) Macular ganglion cell–inner plexiform layer (mGCIPL) ratio versus macular vessel density (mVD) ratio. Linear regression lines with shaded 95% confidence intervals are shown separately for each group.

Correlation analysis between structural and vascular ratios was performed separately for each group (Fig 3 and S1 Table). In the NAION group, a strong positive correlation was observed between the mGCIPL and mVD ratios (ρ = 0.815, *p* = 0.001), whereas the correlation between pRNFL and pVD ratios was not statistically significant (ρ = 0.394, *p* = 0.205). In the RTSD group, both mGCIPL and mVD ratios (ρ = 0.469, *p* = 0.021) and pRNFL and pVD ratios (ρ = 0.359, *p* = 0.085) showed moderate positive correlations, although only the macular association reached statistical significance.

To compare the relationship between structural and vascular changes in the macula across groups, an age-adjusted ANCOVA was performed using a linear regression model. In the NAION group, the regression slope between the mGCIPL ratio and mVD ratio was 1.37 (95% CI: [0.78, 1.96], *p* = 0.001), indicating that for every 1% decrease in the mGCIPL ratio, the mVD ratio decreased by approximately 1.37%. In the RTSD group, the regression slope was 0.54 (95% CI: [0.16, 0.92], *p* = 0.007). By comparison, the RTSD group exhibited a reduced slope of 0.75 (95% CI: [0.01, 1.51], *p* = 0.049). These findings suggest that although both groups exhibited a positive association between macular structural and vascular integrity, NAION eyes demonstrated a more substantial reduction in vessel density for a given degree of structural loss compared to RTSD eyes.

## Discussion

This study demonstrated that both RTSD and NAION eyes exhibit significant structural and vascular alterations in retinal regions corresponding to their respective VF defects. Although a positive correlation between structural and vascular parameters was observed in both groups, eyes with NAION showed significantly greater reductions in macular vessel density compared to those with RTSD. These findings indicate that the extent and pattern of retinal vascular involvement differ between RTSD and NAION.

Previous studies have characterized structural and vascular changes in NAION, which is well known to involve primary ischemic damage to both neural and vascular components of the optic nerve head. OCT and OCTA have revealed consistent findings in NAION, including thinning of the peripapillary RNFL and macular GCIPL, as well as marked reductions in peripapillary and macular vessel density that often correlate with visual field loss [8,9,15]. In contrast, research on RTSD using OCTA remains extremely limited. Only a few case reports and small observational studies have suggested possible vessel density reduction in RTSD [11,12], and the extent and consistency of these vascular changes have not been systematically investigated. Although the sample size remains modest, this study includes one of the largest RTSD OCTA datasets reported to date, enabling a structured evaluation of vascular alterations in this underexplored condition. A particular strength lies in the methodological approach, which employed topographically defined affected regions based on visual field defects and compared them with anatomically matched internal control regions from the fellow eye. This within-subject comparison minimized interindividual variability and allowed for precise quantification of structural and vascular changes. To our knowledge, this is the first study to apply a region-specific, internally controlled OCTA analysis to directly compare RTSD and NAION eyes, thereby highlighting distinct patterns of neurovascular involvement in secondary versus primary optic neuropathies.

The distinct patterns of vascular involvement observed in RTSD and NAION likely reflect their fundamentally different pathophysiology. NAION is characterized by a primary ischemic insult, resulting in acute disruption of microvascular perfusion at the level of the optic nerve head and peripapillary retina. This leads to simultaneous axonal loss and capillary dropout, both readily detected using OCT and OCTA. In contrast, RTSD is a secondary degenerative process arising from postgeniculate visual pathway injury, which induces trans-synaptic retrograde degeneration of retinal ganglion cells. The vascular alterations seen in RTSD are likely secondary to decreased metabolic demand following neuronal loss, rather than direct vascular compromise. This mechanism may account for the relatively preserved vessel density in RTSD eyes despite significant structural thinning, in contrast to the more profound vascular dropout observed in NAION.

These differences offer important insight into the interpretation of OCTA findings across a spectrum of optic neuropathies. In RTSD, where the primary lesion is remote from the retina, the vascular changes may represent the baseline level of secondary vascular attenuation attributable solely to neuronal degeneration [12,16]. As such, the RTSD pattern may serve as a physiological reference for distinguishing secondary vascular loss from primary microvascular injury. In optic neuropathies with uncertain or mixed etiology, disproportionate reductions in vessel density relative to structural loss—such as those seen in NAION—may suggest a primary vascular component. Conversely, modest vascular compromise accompanying advanced axonal degeneration may indicate a secondary process.

Unlike conventional fundus photography, which provides a two-dimensional surface view of retinal morphology, OCT angiography enables depth-resolved visualization of the retinal and choroidal microvasculature. This allows quantitative assessment of capillary density and perfusion, revealing subtle microvascular alterations that are not apparent on fundus imaging. Such vascular information complements structural OCT parameters and helps differentiate neural from vascular mechanisms of damage in optic neuropathies.

Particularly in this study, quantifying the structure–vascular relationship through topographically defined, internally controlled OCTA analysis, this study provides a framework for evaluating neurovascular involvement in optic nerve disorders. Such an approach may enhance the interpretation of OCTA findings beyond descriptive metrics, offering potential value in both clinical assessment and pathophysiological investigation.

Despite the strengths of this study—including its use of topographically defined regions, internal control normalization, and comparative analysis between pathophysiologically distinct optic neuropathies—several limitations should be noted. First, the sample size was relatively small, particularly in the NAION group, which may limit the generalizability of the findings. Second, the retrospective design and variability in time since disease onset may have introduced heterogeneity in the degree of structural and vascular degeneration. Although the inclusion criterion of at least four years since lesion onset aimed to minimize this effect in RTSD patients, residual variability cannot be excluded. Third, although individuals with known systemic or ocular conditions that could affect retinal microvasculature (e.g., diabetes mellitus, uncontrolled hypertension, retinal vascular occlusion, glaucoma, or prior ocular surgery) were excluded, the influence of subtle or unrecognized systemic vascular factors cannot be entirely ruled out. Lastly, although topographic alignment was carefully applied when defining the affected and control regions, the sectoral divisions used for OCT and OCTA measurements are not perfectly identical due to inherent differences in each device’s acquisition geometry and analytical design. Our analysis was therefore aligned at the hemiretinal level, corresponding to the functional VF distribution. This approach enables physiologically meaningful comparison between structural and vascular parameters without requiring pixel-level matching. Nevertheless, minor spatial mismatches may persist as an intrinsic limitation of multimodal imaging.

## Conclusion

In conclusion, this study demonstrates that while both RTSD and NAION involve structural and vascular changes detectable on OCT and OCTA, the degree and pattern of vascular compromise differ significantly. NAION is associated with more severe and proportionate vascular attenuation, consistent with a primary ischemic mechanism, whereas RTSD shows more modest vascular changes likely secondary to neuronal loss. These findings underscore the potential of OCTA-based structure–vascular analysis to distinguish between primary and secondary mechanisms of optic nerve injury and provide a comparative framework for interpreting OCTA findings across diverse optic neuropathies.

## Supporting information

S1 Table Correlation analysis between functional outcomes, OCT and the OCTA parameters. (DOCX)

S2 Dataset De-identified dataset of optical OCT and OCTA parameters of study participants. (XLSX)
