# Supplementary material for: Topographic analysis of vascular changes in retrograde trans-synaptic degeneration
Source: PLoS One. 2025 Dec 2;20(12):e0337283. doi: 10.1371/journal.pone.0337283 (PMC12671765; doi:10.1371/journal.pone.0337283)
Supplement: S1 Table — (DOCX) [file pone.0337283.s001.docx]

**Supplementary Table S1. Correlation analysis between functional outcomes, OCT and the OCTA parameters.**

|  | RTSD (n = 24 eyes) | | | | NAION (n= 12 eyes) | | | |
| --- | --- | --- | --- | --- | --- | --- | --- | --- |
|  | pVD ratio | | mVD ratio | | pVD ratio | | mVD ratio | |
|  | ρ | *p*-value | ρ | *p*-value | ρ | *p*-value | ρ | *p*-value |
| BCVA | 0.421 | **0.040** | 0.250 | 0.239 | 0.009 | 0.987 | -0.135 | 0.675 |
| VFI | 0.037 | 0.865 | -0.077 | 0.720 | 0.853 | **<0.001** | 0.315 | 0.319 |
| MD | 0.030 | 0.888 | -0.130 | 0.544 | 0.761 | **0.004** | 0.049 | 0.879 |
| pRNFL ratio | 0.359 | 0.085 |  |  | 0.394 | 0.295 |  |  |
| mGCIPL ratio |  |  | 0.469 | **0.021** |  |  | 0.815 | **0.001** |

RTSD, retrograde trans-synaptic retinal degeneration; NAION, non-arteritic anterior ischemic optic neuropathy; pVD, peripapillary vessel density, mVD, macular vessel density; BCVA, best corrected visual acuity; VFI, visual field index; MD, mean deviation; pRNFL, peripapillary retinal nerve fiber layer; mGCIPL, macular ganglion cell-inner plexiform layer
